# Hospital Dispensers and the Insurance Act

**Published:** 1912-05-11

**Authors:** 


### Hospital Dispensers and the Insurance Act.

*To the Editor of* The Hospital.

Sir,—With reference to your interesting note on "Hospital Dispensers and the Insurance Act," I think there can be no doubt that the Pharmaceutical Standing Committee on Insurance would benefit by the inclusion of a representative of institution dispensers. The specialised knowledge and experience of the institution dispenser would be of service on this Committee and should be utilised to the utmost in the interests of the public and of pharmacy.

If the Committee is to be really representative of all sections in pharmacy, the claim of institution dispensers for representation cannot be passed over.—Yours faithfully,
Public Pharmacist.

May 7.

